# Sex Difference in Network Topology and Education Correlated With Sex Difference in Cognition During the Disease Process of Alzheimer

**DOI:** 10.3389/fnagi.2021.639529

**Published:** 2021-06-03

**Authors:** Xiaoshu Li, ShanShan Zhou, Wanqiu Zhu, Xiaohu Li, Ziwen Gao, Meiqin Li, Shilei Luo, Xingqi Wu, Yanghua Tian, Yongqiang Yu

**Affiliations:** ^1^Department of Radiology, The First Affiliated Hospital of Anhui Medical University, Hefei, China; ^2^Department of Neurology, The First Affiliated Hospital of Anhui Medical University, Hefei, China

**Keywords:** Alzheimer’s disease, cognitive reserve, modularity, network topology, sex difference, small-world property

## Abstract

**Background**: Alzheimer’s disease (AD) manifests differently in males and females. However, the neuro-mechanism and influence factors are still unknown.

**Objective**: To explore sex differences in brain network topology during AD disease progression and its association with cognition and possible influencing factors.

**Methods**: Resting-state functional magnetic resonance imaging (MRI) data and cognitive scores were collected from 82 AD patients (50 females), 56 amnestic mildly cognitive impaired patients (29 females), and 63 healthy controls (38 females). Global and regional topological network metrics and modular architecture were calculated. Two-way ANOVA was performed to explore group and sex interactions and their main effects. Mediation analysis was used to explore the relationship among education, inter/intra-network connectivity, and the Mini-Mental State Examination (MMSE) score.

**Results**: Lower levels of education, lower MMSE scores, and a positive correlation between the level of education and MMSE scores were found in female AD patients (*p* = 0.024, *r* = 0.319). Significantly lower connectivity strength within the sensorimotor network, dorsal attention network, ventral attention network (VAN), and between the sensorimotor and VAN were observed in male AD patients (*p* = 0.006, 0.028, 0.046, and 0.013, respectively). Group and sex interactions were also found in nodal properties, mainly in the frontal lobe, temporal lobe, middle cingulum, precuneus, and postcentral gyrus. Several of the altered brain network properties were associated with cognitive behavior in male AD patients. Education regulated the MMSE score through the mediation of connection strength between the default mode and limbic networks (LN) in the patient group (aMCI and AD combined).

**Conclusion**: Our results demonstrate that sex differences exist at the brain network level in AD. Sex differences in network topology and education are correlated with sex differences in cognition during AD progression.

## Introduction

Alzheimer’s disease (AD) is the most common type of dementia in senile individuals, characterized by progressive and irreversible decline of cognition (Ewers et al., 2011; Scheltens et al., 2016). It is expected to affect 90 million people worldwide by 2050, and has been recognized as a public health priority by the WHO. Notably,AD seems to disproportionately affect women, as demonstrated by epidemiological surveys showing that the prevalence of AD is higher in women than in men (Mazure and Swendsen, 2016). If AD sex specific diagnosis, treatment, intervention, and prevention can be achieved, it may help to alleviate the current bottle-necked AD situation.

Recently, sex differences in AD and its pre-diagnosis stages [such as amnestic mild cognitive impairment (aMCI) and subjective cognition decline (SCD)] have attracted increasing attention. Besides, the higher prevalence of females with AD, cognitive decline was also found to be more rapid in females than in males. An 8 year longitudinal study showed that cognitive deterioration in females with aMCI was twice as fast as for males (Lin et al., 2015). In addition, the annual conversion rate of female aMCI patients to a full AD diagnosis was 2%–3% higher than that of male patients (Tifratene et al., 2015). Sex differences also exist in cognitive ability. Studies have shown that, even under the same conditions of hippocampal atrophy and temporal lobe glucose metabolism rate, the verbal memory performance is different between male and female aMCI patients (Sundermann et al., 2016). There are also sex differences in neuropsychiatric symptoms related to AD. Male AD patients are more likely to exhibit apathy, agitation, and abusive and socially inappropriate behavior, while female patients are more prone to depression, mood instability, and affective symptoms (Mazure and Swendsen, 2016; Ferretti et al., 2018). Why and how do those sex differences occur? Research is urgently needed to investigate the underlying mechanisms and influencing factors of the above-mentioned sex dependent manifestation.

However, the neuro-mechanisms involved in the sex related differences in AD are still unknown. Only a few studies have found that there were sex differences in the rate of brain atrophy in AD. The brain atrophy rate of female patients with aMCI and AD is larger than that of male patients, with an additional decrease of 1.0%–1.5% per year (Ardekani et al., 2016). Female patients with positive AD biomarkers (Aβ and tau) show faster rates of hippocampal atrophy and cognitive decline than male patients (Koran et al., 2017). However, recent research has found that, compared with women, males with SCD showed higher anterior cingulate cortex amyloid load and glucose hypo-metabolism in the precuneus, posterior cingulate, and inferior parietal cortices, as well as lower resting-state functional connectivity (FC) in the default mode network (Cavedo et al., 2018). These results seem discrepant, and more comprehensive research is needed.

One comprehensive way is to model the human brain as a complex network. The combination of non-invasive magnetic resonance imaging (MRI) techniques and graph theoretical approaches enable us to map human structural and FC patterns at the macroscopic level (Bullmore and Sporns, 2009; Liao et al., 2017). Modeling the human brain as a complex network has provided a powerful mathematical framework and a comprehensive understanding of the structural and functional architectures of the brain (Liao et al., 2017). One of the most influential properties of human brain networks is the prominent small-world organization. Such a network architecture in the human brain facilitates efficient information segregation and integration at low wiring and energy costs, which presumably results from natural selection under the pressure of a cost-efficiency balance (Fan et al., 2011; Liao et al., 2017). Another important property of the brain network is modularization. Modules derive from a decomposition of the network into sub-components that are internally strongly coupled, but externally only weakly coupled (Sporns and Betzel, 2016). Methods for detecting modules (also called network communities) are of particular interest because modular network organization plays a crucial role in maintaining the balance between functional specialization and integration, which supports individual cognitive and behavioral capacities (Crossley et al., 2013; Bertolero et al., 2015; Sporns and Betzel, 2016; Ma et al., 2020). Accumulating evidence suggests that this balance is disrupted in AD patients due to dysconnectivity within and between functional modules involving both high-order and primary networks (Dai and He, 2014; Dai et al., 2015, 2019). However, to date, relatively little is known regarding changes of modularized patterns between male and female AD patients. To the best of our knowledge, this is the first work to study sex differences in network modularization during AD progression.

The main purposes of this study were: (1) to explore sex differences in brain network topology during AD progression; (2) to explore the association between sex differences in network topology and cognition; and (3) to explore the possible influencing factors of sex differences during AD progression. We hypothesized that sex differences would exist at the brain network level, and correlate with sex differences in cognition during AD progression.

## Materials and Methods

### Participants

In total 201 right-handed participants took part in this study: 82 AD patients (50 females), 56 aMCI patients (29 females), and 63 normal healthy controls (HCs, 38 females). The detailed demographic baseline information can be found in Table 1. The AD and aMCI patients were recruited from the Dysmnesia Outpatient Department at the 1st Affiliated Hospital of Anhui Medical University, Anhui Province, China. The HCs were recruited from the local community or were the spouses of the patients in the study. All experimental procedures were approved by the Medical Research Ethics Committee of the 1st Affiliated Hospital of Anhui Medical University. All subjects gave written informed consent in accordance with the Declaration of Helsinki.

**Table 1:** Demographics and cognitive features of participants.

	HC-Female	HC-Male	aMCI-Female	aMCI-Male	AD-Female	AD-Male	*P*-value-group	*P*-value-sex	*P*-value-interaction
Number	38	25	29	27	50	32	0.516		
Age	63.76 ± 7.13	67.28 ± 9.12	66.03 ± 8.09	65.07 ± 7.16	65.08 ± 10.34	68.84 ± 9.21	0.539	0.098	0.251
Education years	10.16 ± 3.36	12.52 ± 4.03	8.66 ± 4.75	11.52 ± 4.43	5.98 ± 4.35	9.25 ± 4.39	<0.001**	<0.001**	0.821
TIV	1315.6 ± 92.2	1427.1 ± 96.4	1305.6 ± 84.2	1435.3 ± 73.1	1255.5 ± 85.8	1412.5 ± 84.6	0.016*	<0.001**	0.300
Mean-FD	0.173 ± 0.104	0.175 ± 0.116	0.196 ± 0.105	0.183 ± 0.090	0.180 ± 0.104	0.212 ± 0.125	0.477	0.651	0.479
MMSE	28.66 ± 1.10	28.72 ± 0.98	26.45 ± 1.48	26.41 ± 1.69	15.10 ± 5.60	17.97 ± 4.82	<0.001**	0.063	0.025*
AVLT (Immediate)	9.24 ± 1.70	8.41 ± 1.62	6.34 ± 2.08	6.05 ± 1.67	3.75 ± 1.92	2.69 ± 1.89	<0.001**	0.006**	0.481
AVLT (Delay)	11 (4.25)	9 (4)	6 (3.5)	5 (3)	0 (5.20)	0 (1.75)	<0.001**	0.012*	0.833
AVLT (Recognition)	14.39 ± 0.82	13.76 ± 1.20	13.21 ± 2.19	11.78 ± 2.31	11.23 ± 2.99	8.41 ± 4.96	<0.001**	<0.001**	0.070
DS (Forward)	7.44 ± 1.39	7.87 ± 1.14	7.13 ± 1.38	7.32 ± 1.45	5.67 ± 1.55	5.96 ± 1.99	<0.001**	0.173	0.910
DS (Backward)	4.84 ± 1.41	5.27 ± 1.28	3.61 ± 0.90	4.32 ± 1.33	2.76 ± 1.24	2.90 ± 0.99	<0.001**	0.016*	0.410
VFT	17 (6.65)	16 (6.65)	13.35 (4.50)	13.35 (3.00)	11 (6.35)	10.5 (5.35)	<0.001**	0.882	0.644

TIV, total intracranial volume; mean-FD, mean frame-wise displacement; MMSE, Mini-Mental State Examination; AVLT, Auditory Verbal Learning Test; DS, Digit SpanTest; VFT, Vocabulary fluency test; *p* < 0.05 means significant statistic difference (corrected by multiple comparison). * indicates *p* < 0.05; ** indicates *p* < 0.01.

The diagnosis of AD fulfilled the Diagnostic and Statistical Manual of Mental Disorders 4th Edition criteria for dementia and the revised National Institute of Neurological and Communicative Disorders and Stroke/Alzheimer Disease and Related Disorders Association (NINCDS-ADRDA) criteria for possible or probable AD.The inclusion criteria included: insidious onset; clear-cut history of worsening of cognition by report or observation; the initial and most prominent cognitive deficits were evident both historically and by examination, with patients showing amnestic presentation or non-amnestic presentations such as language, visuospatial, and executive dysfunction (McKhann et al., 2011; Li et al., 2016); the Mini-Mental State Examination (MMSE) score was less than 24; and the Clinical Dementia rating (CDR) score ranged from 0.5 to 2. The exclusion criteria were: history of sudden onset, early occurrence of gait disturbances, seizures and behavioral changes; focal neurological features including hemiparesis, sensory loss, and visual field deficits; early extra-pyramidal signs and other severe disorders such as trauma, major depression, severe cerebrovascular disease, and toxic and metabolic abnormalities (Dubois et al., 2007; Li et al., 2016).

aMCI was diagnosed according to the recommendations from the NINCDS-ADRDA criteria: complaints of memory loss and/or other cognitive changes recognized by the patient, family, or physician, in comparison with the patient’s previous level; evidence of lower performance in one or more cognitive domains that was greater than would be expected for the patient’s age and educational background; preservation of independence in functional abilities and not demented (Albert et al., 2011; Li et al., 2016); an MMSE score >24; and a CDR score of 0.5. The exclusion criteria were the same as the AD patients.

The HCs were identified as cognitively normal with no neurological or psychiatric disorders and who were not taking any psychoactive medication. All HCs had an MMSE score of 28 or higher and a CDR score of 0. The exclusion criteria were the same as the AD and aMCI patients.

### Cognitive Assessment

For each participant, we used the MMSE to determine general cognitive function, the auditory verbal learning test (AVLT) to test episodic memory function, the vocabulary fluency test (VFT) to evaluate linguistic function, and the digital scale (DS) to assess executive function.

### MRI

MRI scans were performed on a General Electric 750 w 3.0 T MRI scanner with a 24-channel head coil (General Electric, Waukesha, WI, USA). The imaging protocol included a T1-weighted three-dimensional structure sequence, resting-state blood oxygen level-dependent (BOLD) functional MRI sequence, axial T2-weighted, and fluid-attenuated inversion recovery images (Li et al., 2020).

T1-weighted three-dimensional structural data were collected using the brain volume sequence [repetition time (TR)/echo time (TE)/inversion time = 8.5/3.2/450 ms; matrix = 256 × 256; field of view (FOV) = 256 mm × 256 mm; slice thickness = 1.0 mm without intervals]; the acquisition time was 4 min 56 s. BOLD data were collected using a gradient single-shot echo planar imaging sequence (TR/TE/flip angle = 2,000 ms/30 ms/90°; matrix = 64 × 64; FOV = 220 mm × 220 mm; 185 volumes; 35 interleaved axial slices with slice thickness of 3 mm and interval space of 1 mm);the acquisition time was 6 min 10 s.

### Resting-State Functional MRI Data Preprocessing

BOLD data were preprocessed using Data Processing Assistant for Resting-state fMRI (DPARSF) software v.3.2^1^ (Yan et al., 2016). The processing pipeline included slice timing, realignment, normalization, nuisance covariate regression, and band-pass filtering. The first 10 volumes for each participant were discarded to allow participants to adapt to scanning noise and for the signal to reach equilibrium. The remaining 175 volumes were corrected for acquisition time delay between slices. Realignment was then conducted to correct the motion between time points. The frame-wise displacement (FD), which indexes volume-to-volume changes in head position, was also calculated. Images were normalized to MNI space using the DARTEL technique and resampled into a 3-mm cubic voxel. Then, nuisance covariates (Friston 24 parameters and white matter and cerebrospinal fluid signals) were regressed out from the data, and spike volumes where the FD exceeded 0.5 mm were also regressed out in order to minimize the influence of head movement. A recent study has demonstrated that strategies without global signal regression enhanced the sensitivity of the detection of differences between AD and control groups in small-worldness and modularity (Chen et al., 2018). Thus, we did not regress out the global signal in our data preprocessing. Datasets were then band-pass filtered in the frequency range of 0.01–0.08 Hz.

### Network Construction and Modular Architecture

The GRETNA software v2.0^2^ was used to perform whole-brain functional network analyses (Wang et al., 2015). A commonly used FC-based atlas (shen-268 atlas) was chosen to calculate the correlation matrix (Shen et al., 2013; Finn et al., 2015). Since we mainly focused on cerebrum function, we removed the cerebellum and brain stem from the shen-268 atlas, which resulted in 218 identified brain regions. Then, we calculated the correlation coefficient between each remaining region to get a 218*218 FC matrix. The FC matrix was then fisher-Z transformed.

Next, we calculated both global and regional topological metrics for each participant. The global network metrics included local efficiency (E_loc_, a measure of the fault tolerance of the network, reflecting how well the information is communicated within the neighbors of a given node when this node is eliminated), global efficiency (E_glob_, a measure of the global efficiency of parallel information transfer in the network), and five small-world property metrics. The small-world property metrics included the clustering coefficient (Cp, a measure of the extent of local density or cliquishness of the network, which reflects network segregation), characteristic path length (Lp, a measure of the extent of average connectivity or overall routing efficiency of the network, which reflects network integration), normalized clustering coefficient (γ, the ratio of clustering coefficients between real and random networks), normalized characteristic path length (λ, the ratio of the characteristic path lengths between real and random networks), and small-worldness property (σ, σ = γ/λ, a scalar quantitative measurement of the small-worldness of a network). For the regional network metrics, we evaluated nodal clustering coefficient (Cp_nodal_), nodal degree centrality (D_nodal_), nodal betweenness (B_nodal_), nodal efficiency (E_nodal_), and nodal local efficiency (Le_nodal_; Li et al., 2020). Binary graph methods for global and regional topological metrics were used, as they were computationally straightforward and provided for simpler interpretation. We applied a range of sparsity thresholds (range of 0.05–0.50 with an interval of 0.05) to ensure the generated networks were estimable for small-worldness and had sparse properties with the minimum number possible of spurious edges. The number of random networks was set as 100.

For modular architecture, the whole-brain functional networks were parcellated into eight modules using a predefined cortical parcellation to reduce disorder bias (Yeo et al., 2011). The eight modules were the default-mode network (DMN), frontoparietal network (FPN), sensorimotor network (SMN), visual network (VN), dorsolateral attention network (DAN), ventral attention network (VAN), limbic network (LN), and subcortical network (SN). The inter-modular connection strength for every pair of modules and the intra-modular connection strength for each module were calculated. Although there is an ambiguous meaning of negative connectivity, some previous studies (Kaiser et al., 2015; Harrison et al., 2016) have demonstrated inverse activity of networks in AD, so for modular architecture, we calculated both positive and negative connectivity in the current study.

### Statistical Analysis

Statistical analysis was conducted using SPSS (version 26.0) software and in-house script on matlab. The chi-square test was used to test sex composition in each group. For data that did not satisfy normal distribution or equal variance, the rank sum test was used. Two-way analysis of variance (ANOVA) was performed to explore group (i.e., HC, aMCI, and AD) and sex (i.e., female, and male) interactions and their main effects in our study. In the statistical model, group and sex were treated as fixed factors. The age, level of education, total intracranial volume, and mean FD were used as covariates. Correlation analysis was performed between network topological properties and cognitive function scores. If the data met the requirements of normal distribution and variance homogeneity, Pearson’s correlation was conducted, otherwise Spearman’s correlation was conducted. We also performed the mediation analysis to further elucidate relationship among education, brain function, and cognition.

### Verification Analysis

In order to avoid the template atlas influence and to test the stability of the results, we also performed a verification analysis. Specifically, we used a further two atlases, AAL90 (Tzourio-Mazoyer et al., 2002) and Craddock200 (Craddock et al., 2012), and repeated the entire analysis for each atlas.

## Results

### Demographic and Clinical Characteristics

Demographic and Clinical Characteristics were summarized in Table 1. Notably, females had lower levels of education than males, especially in AD patients (mean_female_AD__ = 5.98, mean_male_AD__ = 9.25, *p* < 0.001). Females showed better episodic memory function (reflected by AVLT scores) than males, whereas males showed better executive function (reflected by DS backward scores) than females. It is worth noting that the general cognitive function (reflected by MMSE scores) showed an interaction effect of group and sex (*p* = 0.025). In aMCI patients, MMSE scores decreased equally in males and females, however, in AD patients, MMSE scores decreased more quickly in females than in males. Female AD patients had lower MMSE scores than male patients (*p* = 0.016). Notably, a positive correlation between education and MMSE was detected in female AD patients (*p* = 0.024, *r* = 0.319). During AD progression,we also found positive correlations between education and cognitive scores in both male and female patients. In male patients (aMCI and AD combined), education was positively correlated with MMSE, AVLT immediate, and DS forward and backward scores (*p* = 0.039, 0.028, 0.004 and 0.007, *r* = 0.270, 0.287, 0.367 and 0.350 respectively). In female patients (aMCI and AD combined), education was positively correlated with MMSE and DS backward scores (*p* < 0.001, *r* = 0.400 and *p* = 0.009, *r* = 0.291, respectively).

### Modular Properties of Brain Networks

#### Interaction Effects of Group and Sex

**Figure 1:**
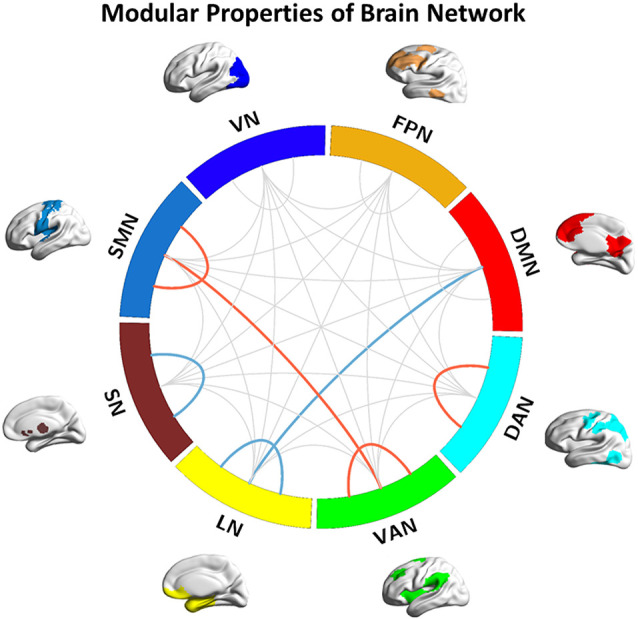
Modular properties of the brain network. Red lines represent the interaction effects of group and sex (detected both intra- and intermodular); Blue line represent decreased inter- and intramodular connection strength in Alzheimer’s disease (AD) patients. DMN, default-mode network; FPN, fronto-parietal network; SMN, sensorimotor network; VN, visual network; DAN, dorsolateral attention network; VAN, ventral attention network; LN, limbic network; SN, subcortical network.

**Figure 2:**
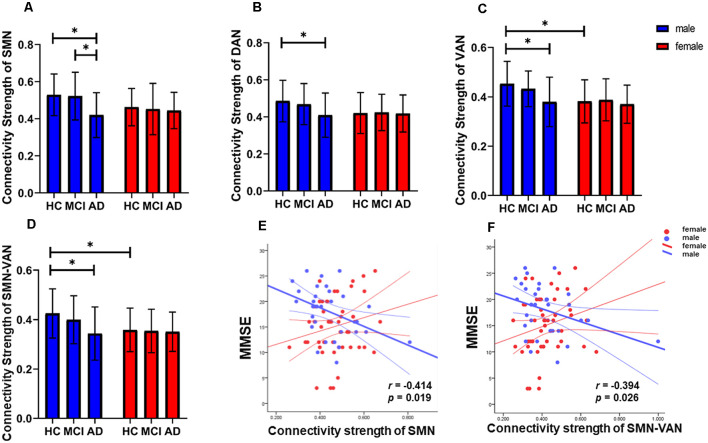
Detailed interaction effects of group and sex in brain modular architecture (*p* < 0.05). **(A–C)** The interaction effect of group and sex within the SMN, DAN, and VAN. **(D)** The interaction effect of group and sex between the SMN and VAN. **(E)** The connection strength of the intra-SMN was negatively correlated with Mini-Mental State Examination (MMSE) scores in male AD patients. **(F)** The connection strength between the SMN and VAN was negatively correlated with MMSE scores in male AD patients. *Indicates significantly different. SMN, sensorimotor network; DAN, dorsolateral attention network; VAN, ventral attention network.

We observed significant interaction effects of group and sex on the connection strength within the SMN, DAN, and VAN, and the connection strength between SMN and VAN (*p* = 0.006, 0.028, 0.046, and 0.013, respectively, Figure 1), indicating different disease trajectories in female and male patients. Specifically, in females, the connection strength within the SMN, DAN, and VAN, and between the SMN and VAN remained unchanged among the groups. In contrast, in males, the connection strength within the SMN was significantly lower in the AD group than in the HC and aMCI groups (*p* = 0.009 and *p* = 0.013, respectively, Figure 2A). Compared with the HCs, only male AD patients showed decreased connection strength within the DAN and VAN, and between SMN and VAN (*p* = 0.038, 0.026 and 0.016 respectively, Figures 2B–D). The connection strength within VAN, and between SMN and VAN, was significantly higher in males than in females in the HC group (*p* = 0.003 and *p* = 0.016, respectively), whereas no significant differences were found in the aMCI and AD groups (Figures 2C,D). Correlation analysis showed that the connection strength within SMN and the between SMN and VAN were negatively correlated with MMSE scores in male AD patients (*p* = 0.019, *r* = −0.414, and *p* = 0.026, *r* = −0.394, respectively, Figures 2E,F).

#### Main Effects of Group

Compared with the HC group, decreased inter-modular connection strength between the DMN and LN, and decreased intra-modular connection strength within the SN, were found in the AD group (*p* = 0.037 and *p* = 0.047, respectively, Figure 1). Compared with the aMCI group, decreased intra-modular connection strength in the LN was found in the AD group (*p* = 0.047, Figure 1). There were no significant inter- or intra-modular connection strength changes in the aMCI group. Correlation analysis showed that the positive connection strength between the DMN and LN was positively correlated with AVLT (delayed scores) in AD patients (*p* = 0.044, *r* = 0.223).

#### Mediation Analysis

**Figure 3:**
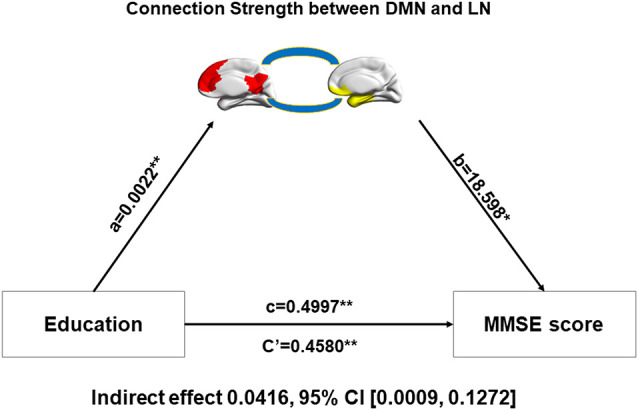
Relationship among education, inter-network connectivity, and the MMSE score. Mediation analysis between education (X) and MMSE score (Y), with DMN-LN connectivity strength as the mediator. Path coefficients with *p* values (*p < 0.05 and **p < 0.01, respectively). DMN, default-mode network; LN, limbic network.

Decreased modular connection strength between and within networks, but better MMSE scores, were detected in male AD patients, which seemed to be intuitively paradoxical. We speculated that cognitive reserve may play an important role in this paradoxical phenomenon. In order to verify our speculation,we performed the mediation analysis in patient group (aMCI and AD combined). The dependent variable was the MMSE score and the independent variable was the level of education, which was a proxy of cognitive reserve. The mediation variable was the connection strength between or within networks. Age was used as the covariate. We found that education was able to regulate MMSE scores through the mediation of connection strength between the DMN and LN. The indirect effect was 0.0416 and the 95% confidence interval was between 0.0009 and 0.1272 (Figure 3).

#### Global and Regional Topological Metrics of Brain Network Interaction Effects of Group and Sex

For global topological metrics: No interaction effect of group and sex was found.

**Figure 4:**
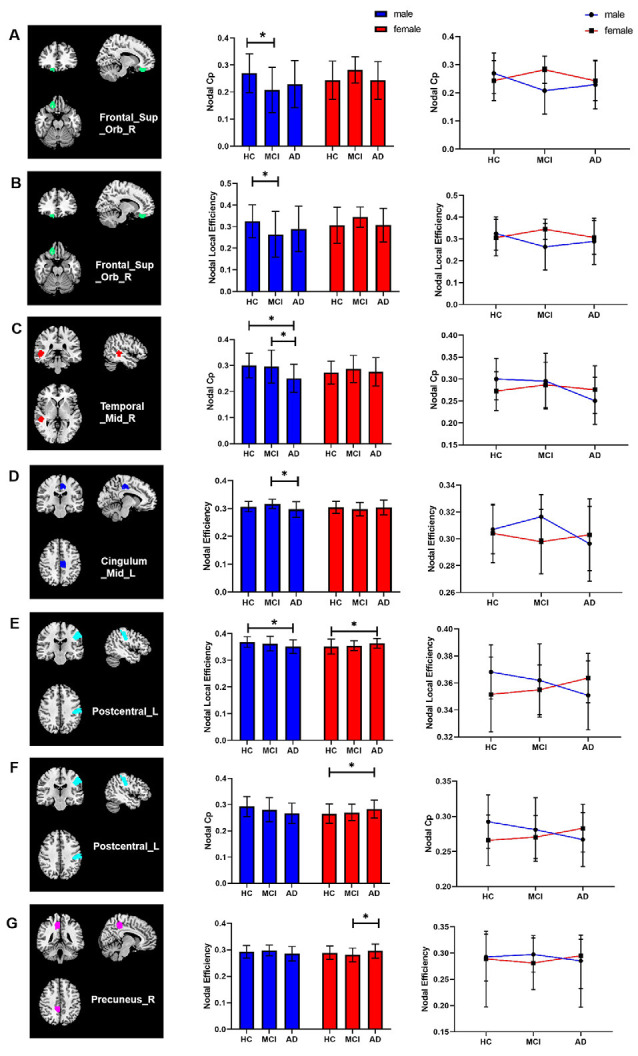
Interaction effects of group and sex in regional topological metrics of brain networks (*p* < 0.05). **(A–E)** Compared with HCs, male aMCI patients showed decreased Cp_nodal_ and Le_nodal_ of the right orbital frontal lobe, whereas male AD patients showed decreased Cp_nodal_ of the right middle temporal gyrus, decreased E_nodal_ of the left middle cingulum, and decreased Le_nodal_ of the left postcentral gyrus compared with HCs and aMCI patients. **(E–G)** Compared with HCs, the Le_nodal_ and Cp_nodal_ of the left postcentral gyrus was increased in female AD patients. Compared with aMCI patients, the E_nodal_ of the right precuneus was significantly increased in female AD patients. *p < 0.05.

For regional topological metrics: the Cp_nodal_ of the right superior orbital frontal gyrus, left postcentral gyrus, and right middle temporal gyrus, the E_nodal_ of the left middle cingulum and right precuneus, and the Le_nodal_ of the right superior orbital frontal gyrus and left postcentral gyrus showed interaction effects between group and sex (all *p* < 0.05), indicating different disease trajectories in females and males. Compared with HCs, male aMCI patients showed decreased Cp_nodal_ and Le_nodal_ of the right orbital frontal lobe, whereas male AD patients showed decreased Cp_nodal_ of the right middle temporal gyrus, decreased E_nodal_ of the left middle cingulum, and decreased Le_nodal_ of the left postcentral gyrus compared with HCs and aMCI patients. Compared with HCs, the Le_nodal_ and Cp_nodal_ of the left postcentral gyrus was increased in female AD patients. Compared with aMCI patients, the E_nodal_ of the right precuneus was significantly increased in female AD patients (Figure 4).

Correlation analysis showed that the Cp_nodal_ of the right superior orbital frontal gyrus was positively correlated with AVLT (recognition score) in male aMCI patients (*p* = 0.042, *r* = 0.427) and that the Cp_nodal_ of the right middle temporal gyrus was positively correlated with AVLT (immediate score and recognition score) in male AD patients (*p* < 0.001, *r* = 0.621 and *p* = 0.041, *r* = 0.388, respectively). The Le_nodal_ of the right superior orbital frontal gyrus was negatively correlated with VFT scores in male AD patients (*p* = 0.004, *r* = −0.523).

#### Main Effects of Group

For global topological metrics: compared with the HCs, the Cp was lower in the AD patients, whereas the gamma and sigma of aMCI patients were higher than that of HCs. For regional topological metrics: compared with the HCs, the Cp_nodal_ of the left middle frontal gyrus decreased in both aMCI and AD patients. Decreased Cp_nodal_ of the right superior parietal gyrus was found in aMCI patients. However, increased E_nodal_ of the left superior temporal pole and increased Le_nodal_ of the left hippocampus were found in aMCI patients, whereas increased E_nodal_ of the right superior parietal gyrus and left middle frontal lobe, but decreased Le_nodal_ of the right middle temporal gyrus, were found in AD patients. When compared with aMCI patients, AD patients showed lower Cp_nodal_ in the right middle temporal gyrus, but higher E_nodal_ in the left caudate nucleus (all *p* < 0.05).

Correlations with cognitive function are summarized in Tables s 2, 3. In AD patients, the Cp of the whole network was positively correlated with both immediate and delayed AVLT scores. The Cp_nodal_ of the left middle frontal gyrus was positively correlated with delayed AVLT scores. The E_nodal_ of the left middle frontal gyrus and left caudate were negatively correlated with immediate AVLT and MMSE scores, respectively. In aMCI patients, the Cp_nodal_ of the right superior parietal gyrus was positively correlated with forward digit span scores and the Cp_nodal_ of right middle temporal gyrus was positively correlated with immediate AVLT scores. The E_nodal_ of the left caudate was negatively correlated with backward digit span scores. Finally, the Le_nodal_ of the left hippocampus was negatively correlated with immediate AVLT scores.

**Table 2:** Correlation analysis with cognition function in AD patients.

	AVLT (immediate)		AVLT (delay)		MMSE	
Brain regions	*r*	*p*	*r*	*p*	*r*	*p*
Cp_whole network_	0.265	0.019	0.270	0.017		
Cp_nodal_						
Frontal_Middle_L			0.255	0.024		
E_nodal_						
Frontal_Middle_L	−0.225	0.048				
Caudate_L					−0.233	0.040

**Table 3:** Correlation analysis with cognition function in aMCI patients.

	AVLT (immediate)		Digit span (forward)		Digit span (backward)	
Brain regions	*r*	*p*	*r*	*p*	*r*	*p*
Cp_nodal_						
Parietal_Superior_R			0.408	0.003		
Temporal_Middle_R	0.283	0.042				
E_nodal_						
Caudate_L					−0.275	0.048
Le_nodal_						
Hippocampus_L	−0.374	0.006			

#### Verification Analysis Results

The results were the same with Shen-268 when using Craddock200 as the atlas. The mediation effect of intra DMN-LN connection strength was also evident. For the AAL90, a portion of results were the same as those obtained with Shen-268, but the interaction effect of group and sex on the connection strength between the SMN and VAN was no longer evident (*p* = 0.071). Likewise, the interaction effect of group and sex on the connection strength of the intra-DAN and VAN was no longer evident (*p* = 0.30 and 0.41).

## Discussion

In the current study, we mainly explored the neuro-mechanism and influence factors of sex differences in AD progression from a brain network perspective. First, we found that sex differences existed in regard to cognitive function. Females showed better episodic memory than males, while males showed better executive function than females. It is worth noting that female AD patients had lower MMSE scores than male AD patients. Second, we found the interaction effects of group and sex existed both in the modular architecture and regional topological metrics of brain networks during AD progression. For modularity manifestation, the sex difference was reflected in decreased connection strength within the SMN, DAN, and VAN, and between SMN and VAN. Compared with HCs and aMCI patients, only male AD patients showed a decreased connection strength within the SMN, DAN, and VAN, and a decreased connection strength between the SMN and VAN. For regional topological metrics, a sex difference was found in the nodal clustering coefficient, nodal efficiency,and local efficiency, mainly in the frontal lobe, temporal lobe,middle cingulum, precuneus, and postcentral gyrus. Third, for the main effects of group, we found a decreased inter-modular connection strength of DMN-LN and an intra-modular connection strength of the LN and SN in AD patients. Compared with HCs, the Cp was lower in AD patients, while the gamma and sigma of aMCI patients were higher. Changes of regional topological metrics were also found in the main effect of group. Fourth, the level of education was higher in male than female patients and correlation analyses showed that education was positively correlated with cognitive scores in both male and female patients. Further mediation analysis confirmed that education was able to regulate MMSE scores through the mediation of connection strength between the DMN and LN in patient group (aMCI and AD combined).

For interaction effects, we found sex specific influences on group. According to our results, the regional topological metrics were increased to some extent during AD progression in female patients. Compared with HCs, the Cp_nodal_ and Le_nodal_ of the left postcentral gyrus was increased in female AD patients. Compared with aMCI patients, the E_nodal_ of the right precuneus was significantly increased in female AD patients. The precuneus is a vital brain region that participates in primary and advanced cognitive activities. We speculate that the increased nodal properties may act as a compensatory mechanism against the pathological damage of AD. For male patients, we found the opposite results. Male AD patients showed a decreased connection strength between the SMN and VAN, and within the SMN, DAN, and VAN. Compared with HCs, male aMCI patients showed a decreased nodal clustering coefficient and nodal efficiency of the orbital frontal lobe, whereas male AD patients showed decreased Cp_nodal_ of the right middle temporal gyrus and decreased E_nodal_ of the left middle cingulum. The topological changes of these brain regions, however, did not occur in female aMCI or AD patients.

The findings seemed intuitively paradoxical to the higher prevalence of AD in women in a previous epidemiologic study, and to that of the higher MMSE scores in male AD patients. One possible explanation is that males may have higher resilience to AD-related brain pathological damage, which is supported by the cognitive reserve theory. Cognitive reserve is postulated to act as a moderator between pathology and clinical outcome, which enables patients to tolerate brain pathological damage and maintain function (Stern, 2002, 2012; Ewers, 2020). Studies have demonstrated that educational and occupational attainment can increase cognitive reserve (Bennett et al., 2003; Hall et al., 2007; Garibotto et al., 2008; Stern, 2012; Yasuno et al., 2020). In the current study, more severe connection damage between the SMN and VAN, and within the SMN, DAN, and VAN were found in male AD patients. The DAN, which is bilaterally centered on the intraparietal sulcus and the frontal eye fields, appears to be involved in the endogenous goal-driven attention orienting (top-down) process and is responsible for the preparation and selection for stimuli and responses. The VAN (which includes the right lateralized temporal-parietal junction and the ventral frontal cortex) is involved in the exogenous stimuli-driven attention reorienting (bottom-up) process and is activated when detecting unexpected salient targets (Li et al., 2012; Qian et al., 2015). The attention networks are commonly impaired in AD patients. The SMN is involved in episodic memory, action recognition, and spatial navigation (Li et al., 2015). The higher levels of education of male patients in our current study may have counterbalanced the decreased topological properties in the brain and helped to maintain function, especially in regions involved in attention resourcing during memory encoding or spatial navigation. Epidemiological studies have suggested that at any given level of clinical severity in AD, the degree of pathology will be greater in individuals with higher cognitive reserve than in those with lower cognitive reserve (Stern, 2012). A recent study, which focused on sex differences in functional and molecular neuroimaging biomarkers of SCD, also found that male SCD patients showed a higher anterior cingulate cortex amyloid load and glucose hypometabolism in the precuneus, posterior cingulate, and inferior parietal cortices, and lower resting-state FC in the DMN (Cavedo et al., 2018), which were consistent with our findings. In contrast, females may have lower resilience to AD-related brain pathological damage and, thus, even during relatively minor changes of modular network organization, and combined with the lower levels of education in the current study, showed worse general cognition behaviors (reflected by MMSE scores). Further mediation analysis also confirmed that the level of education was able to regulate MMSE scores through the mediation of connection strength between the DMN and LN in the patient group (aMCI and AD combined). We speculate that patients with a higher level of education may have a relatively stronger connection strength between the DMN and LN than those with a lower level of education, leading to better MMSE scores. However, since our study was only a cross section study from a brain network perspective, caution is needed when interpreting the results that brain damage of female AD was slightly more than that of male AD patients. Our study supported the cognitive reserve theory, although more studies are needed to explain the relationships among cognitive reserve, brain alterations, and cognitive behaviors, and not only at a functional level, but also at the gray and white matter structure levels.

For main effects, since only considering sex (regardless of disease) does not have great clinical benefit, we placed an emphasis on the main effects of group in this study, which can be understood as the influence of AD itself, regardless of sex. We found lower Cp in AD patients, while higher gamma and sigma were found in aMCI patients, which were consistent with previous studies (Bai et al., 2013; Brier et al., 2014; Dai and He, 2014). For modular architecture, we detected a decreased inter-modular connection strength between the DMN and LN, and decreased intra-modular connection strength of LN and SN in AD patients. The DMN is known to be involved in introspection episodic memory processing, and in monitoring the internal and external environment and stream of consciousness (Barkhof et al., 2014; Li et al., 2016). Many resting-state functional MR studies have demonstrated reduced DMN connectivity in aMCI and AD patients, even in unaffected carriers of familial AD and subjects with cognitive complaints (Greicius et al., 2004; Zhao et al., 2012; Wang et al., 2013; Barkhof et al., 2014; Cai et al., 2017). Reduced integrity in the DMN is associated with amyloid beta and tau pathology before the clinical onset of AD. The LN has been considered to play important roles in memory, emotion, spatial orientation, visceral sensation, and behavior (Catani et al., 2013; Li et al., 2016). An impaired brain LN is closely associated with impaired cognition in AD. In our study, we found a disconnection of the DMN and LN. Later, correlation analysis confirmed that changes of inter-DMN and LN connection strength were associated with decreased cognitive function, especially in episodic memory.

The brain regions with node attribute changes were mainly distributed in the DMN, LN, SMN, SN, and FPN, which overlapped with the results of modularization to some extent, i.e., the results were mutually verified and complemented. Correlation analyses showed close associations between the changed network properties and cognition function, which implied an underlying sex specific neuro-mechanism to cognitive maintenance in male AD patients. Validation tests also confirmed that our results were robust. Different atlases may also influence the results, and we surmise that a more elaborate atlas (such as Shen-268 or Craddock200) may be more suitable in brainnetome analysis, but further studies are required to prove our speculation.

There were several limitations in the current study. First, the sample size was relatively small, which may have limited the detection of significant brain alterations and limited the *p* values to survive in the multiple comparison correction. A larger sample size is therefore warranted in future studies. Second, we did not include measures of AD pathology due to limited conditions, but the patients were screened by comprehensive clinical evaluation and we believe all were placed in the correct groups. Future studies should focus on how AD-related pathology may disrupt brain network organization and how this may result in cognitive changes. Third, in the current study, we preliminarily explored the relationship among the level of education, brain alterations, and MMSE scores; more studies are needed to explain the relationships among cognitive reserve, brain alterations, and cognitive behaviors, not only at a functional level, but also at the gray and white matter structure levels. Fourth, we tested the basic clinical screening scales, but lacked assessments that may be more sensitive to targeting specific networks such as attention and sensorimotor networks. We will employ more specialized and sensitive scales in future work. Fifth, we did not include SCD patients in our current study, and future studies should incorporate SCD patients in the study cohort. Finally, we only conducted a monocentric cross-sectional study; independent site cross-validation and longitudinal studies are warranted in future studies.

In conclusion, the findings of our current study demonstrate that sex differences exist at the brain network level, as does a correlation between sex and cognition during AD progression. The level of education may be an important factor that influences cognition and sex specific brain network topological properties in AD patients. Further studies are needed to explore the precise sex-dependent relationships among cognitive reserve, brain alterations, and cognitive behaviors.

## Data Availability Statement

The raw data supporting the conclusions of this article will be made available by the authors, without undue reservation.

## Ethics Statement

The studies involving human participants were reviewed and approved by the Medical Research Ethics Committee of the first Affiliated Hospital of Anhui Medical University. The patients/participants provided their written informed consent to participate in this study.

## Author Contributions

YY designed the study and revised it critically for important intellectual content. XSL performed the research and drafted the manuscript. WZ, ZG, ML, and SL helped in MRI data collection. XHL helped draft the work. SZ, YT, and XW participated in the clinical evaluation of patients. All authors contributed to the article and approved the submitted version.

## Conflict of Interest

The authors declare that the research was conducted in the absence of any commercial or financial relationships that could be construed as a potential conflict of interest.

## Footnotes

^1^http://rfmri.org/DPARSF

^2^http://www.nitrc.org/projects/gretna
